# An imbalanced learning method based on graph tran-smote for fraud detection

**DOI:** 10.1038/s41598-024-67550-4

**Published:** 2024-07-17

**Authors:** Jintao Wen, Xianghong Tang, Jianguang Lu

**Affiliations:** 1 College of Computer Science and Technology, Guizhou University, Guiyang, 550025 China; 2 State Key Laboratory of Public Big Data, Guizhou University, Guiyang, 550025 China

**Keywords:** Fraud detectiont, Subgraph structure, Feature extraction, Node embedding, Oversampling methods, Computer science, Information technology

## Abstract

Fraud seriously threatens individual interests and social stability, so fraud detection has attracted much attention in recent years. In scenarios such as social media, fraudsters typically hide among numerous benign users, constituting only a small minority and often forming “small gangs”. Due to the scarcity of fraudsters, the conventional graph neural network might overlook or obscure critical fraud information, leading to insufficient representation of fraud characteristics. To address these issues, the tran-smote on graphs (GTS) method for fraud detection is proposed by this study. Structural features of each type of node are deeply mined using a subgraph neural network extractor, these features are integrated with attribute features using transformer technology, and the node’s information representation is enriched, thereby addressing the issue of inadequate feature representation. Additionally, this approach involves setting a feature embedding space to generate new nodes representing minority classes, and an edge generator is used to provide relevant connection information for these new nodes, alleviating the class imbalance problem. The results from experiments on two real datasets demonstrate that the proposed GTS, performs better than the current state-of-the-art baseline.

## Introduction

Fraud detection is one of the crucial areas in today’s digital age, and aims to identify and prevent various forms of fraudulent behavior, such as online scams^1^, health insurance fraud^2^, fake account detection^3^ and opinion audit fraud^4^, etc. Fraud not only causes huge losses to the economy^5^, but also damages user trust, destroys the reputation of organizations, and may even threaten public safety. As technology continues to advance and business activities are digitally transformed, fraudsters are also becoming increasingly ingenious and diverse, and makes fraud detection an area of ongoing challenge. Therefore, there is an urgent need to conduct research on fraud detection methods.

The research on fraud detection methods has a lot of practical significance, and the related research results can be applied to other fields, such as bank card fraud detection^6^ and early warning or transaction fraud detection^7^ and early warning, so as to significantly reduce the economic losses caused by fraud and improve people’s sense of network security.

However, in most fraud detection cases, the suspected fraudulent data typically represents only a small fraction of the overall data. For example, credit card fraud detection^8^, an open-source dataset provided by Worldline and the Machine Learning Group, has a total dataset of 284,807 records, of which only 492 are fraudulent transaction data. YelpChi^9^, a real review dataset from Yelp.com, merely 14.5% of the reviews fall into the category of spam reviews, and the remaining are designated as recommended reviews. Thus, the classes faced by fraud detection are usually extremely unbalanced, and a few classes are crucial for in fraud detection because they may be potential fraudsters.

**Figure 1:**
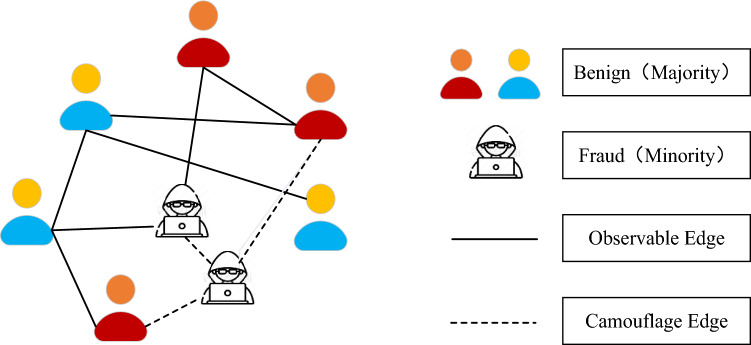
An example of an extreme imbalance between the classes of fraudsters and benign users, where there are six benign users and only two fraudsters. When features is aggregated by GNN, the feature information of fraudsters is easily diluted.

In order to cope with the data imbalance problem, researchers have used various strategies in recent years. Two common ones among these strategies are oversampling^10^ and undersampling^11^. While undersampling accomplishes balance by reducing the number of majority classes, oversampling balances the data by increasing the number of minority classes. However, these strategies have some drawbacks. Oversampling may lead to overfitting and undersampling may lead to loss of information. Another strategy is to use Synthetic Data, which balances the data by synthesizing samples of minority classes. Strategies based on synthesizing minority class data include SMOTE^12^ (synthetic minority over-sampling technique) and GANs^13^ (generative adversarial networks). However, the data synthesized by these strategies are unconnected to each other, and applying them directly to fraud detection is not feasible because fraud data are rich in interactions with each other. An example of class imbalance and behavioral interactions between fraudsters and benign users is shown in Fig. 1.

Currently, in order to cope with the rich behavioral interactions between fraudulent data and the class imbalance problem, fraud detection methods based on graph neural networks (GNN) have been widely studied. Examples include GraphSMOTE^14^, PC-GNN^15^ and OA-GNN^16^. However, they did not predict the types of edges when studying the graph-based class imbalance problem, and a few class nodes may be underrepresented, and due to the attribute of “small gangs” among fraudsters, the increase in the number of aggregation layers may easily lead to oversmoothing phenomenon. Therefore, finding the appropriate number of aggregation layers in the aggregation process is also crucial to improve the model representation capability.

To address the above problems, we propose a graph tran-smote based unbalanced learning method for fraud detection. The nodes and edges of the graph data are feature extracted by graph transformer to make the embedded nodes with structural information. Then SMOTE is used to synthesize the nodes of a few classes in the embedded domain, while the edges are synthesized using the extracted structural information. Finally the classifier is trained for node classification. The main contributions of this paper are as follows:

- Introduced a new subgraph neural network extractor to deeply mine node structural features, preventing over-smoothing and compression during embedding.
- Implemented a transformer-based feature extractor to enhance node information representation by integrating structural and attribute features.
- Developed a feature embedding space for generating minority class nodes and an edge generator for providing connectivity, effectively resolving class imbalance.
- The method’s effectiveness was illustrated through experiments carried out on two authentic public datasets.

The structure of the paper is as follows: in “Related work” section presents related work. In “Methodology” section provides a detailed description of the proposed GTS framework. In “Experiments” section encompasses experiments conducted to assess the effectiveness of GTS. In “Conclusion” section offers a summary of the paper.

## Related work

Fraud detection is a node classification task for behavioral assessment based on user attributes. Numerous GNN-based techniques have been used to tackle the fraud detection task as a result of the diverse behavioral interactions that occur between users^17^. However, directly applying traditional GNN-based methods, such as GCN^18^, GraphSAGE^19^, GAT^20^, etc. They are not effective for fraud detection because there is complex and important structural information between fraudulent data, and traditional GNN-based methods are poorly sensitive to structural information.

Later, in order to improve the sensitivity to fraud data, researchers innovated many models on the traditional GNN models. For example, GraphRAD^21^, GEM^22^, SemiGNN^1^ and H2-fdetector^23^, etc. GraphRAD^21^ is the first time to apply graph neural networks to the fraud detection problem, and it is also the first method to apply local graph clustering to the payment fraud detection problem. But it does not consider the sparsity problem of fraud data. GEM^22^ initially brings heterogeneous graph neural networks into the fraud detection domain. It adaptively acquires distinctive embeddings from diverse account-device graphs to identify malicious accounts effectively. But the authenticity of embeddings is not considered. SemiGNN^1^ obtains unlabeled data by extending the social relations of the labeled data and proposes a semi-supervised attentional graph neural network for fraud detection using multi-view labeled and unlabeled data. DCI^24^ applies the concept of self-supervised learning and comparative learning for the first time on a dataset for fraud detection, and verifies the effectiveness of self-supervised learning for the fraud detection or anomaly detection problem. CARE-GNN^25^ introduces two kinds of camouflage behaviors, namely feature camouflage and relation camouflage. The aggregation process of GNN is enhanced by three unique modules to counteract the artifacts. CIES-GNN^26^ utilizes graph structure information to identify fraud-induced artifacts, and embeds deep entities with rich semantics, using artifact recognition and enhanced semantic aggregation for fraud detection. MAFI^27^ addresses the limitations in expression and challenges in dealing with disguised entities in traditional GNNs for fraud detection. However, by incorporating multiple aggregators and their attention mechanisms, the model significantly increases the demand for computational resources. H2-FDetector^23^ addresses the issue of neglecting heterogeneity in connection differences in fraud detection. But, the performance of the model depends on the quality and quantity of labeled nodes, and inaccuracies or imbalances in labeling can affect the model’s accuracy. LGM-GNN^28^ addresses the issue of overly focusing on local information while neglecting global information. However, this approach may result in high computational complexity and memory requirements, becoming a performance bottleneck when processing large-scale datasets. ASA-GNN^29^ addresses the performance degradation caused by reliance on raw features, manual feature engineering, and imitative behaviors. But, the complex graph structure and adaptive sampling strategies may lead to overfitting, particularly in scenarios with small datasets or uneven data distribution. SCN-GNN^30^ addresses the issues of limited node interconnectivity and sparse information. But, it may lose generalization capability due to overfitting specific training data features. However, the above methods do not take into account the “small gangs” property of fraud detection, which leads to oversmoothing in the aggregation process. At the same time, the class imbalance problem of fraud data is not taken into account.

Several studies have been devoted to the problem of class imbalance on graphs. Fraudre^31^ encodes the original node features to apply a loss function to the imbalanced classification module. However, it does not really solve the class imbalance problem for fraudulent data. GraphSMOTE^14^ constructs an embedding space to encode the similarity between nodes, while training edge generators to model the relational information and provide associative information for new samples. However, it does not consider the structural information in relation to the nodes. Boosting-GCN^32^ introduces a unified model that employs a gnn as the base classifier during the enhancement procedure. It assigns greater weights to training samples that are not accurately categorized by preceding classifiers. DR-GCN^33^ proposes a class-conditional adversarial training process to facilitate the separation of labeled nodes. GNN-CL^34^ proposes adaptive graph oversampling and neighborhood-based dynamic sampling operations and loss backpropagation for metric learning. PC-GNN^15^ first picks nodes and edges with a designed label-balanced sampler to construct subgraphs for small batch training. But it does not consider the global imbalance of classes. AO-GNN^16^ solves the label imbalance problem in GNN by maximizing the loss function of AUC. However, this may increase the computational complexity and lead to poor generalization. HHLN-GNN^35^ addresses the issues of high imbalance and complexity in transaction data as well as the insufficient handling of the stealthy behavior of fraudulent entities. But, the model may not adequately capture the subtle behaviors of fraudulent transactions that do not follow typical patterns. Cost-Sensitive^36^ addresses the issue of graph imbalance in mobile social network fraud detection. However, integrating multiple complex steps may result in high computational costs and time consumption.

Similarly, the above methods don’t take into account the “small gangs” property of fraud detection and ignore the important impact of structural information on the representation of node features.

## Methodology

In this section, the proposed GTS methodology will be described in detail. First, the motivation of the proposed model will be described. Then, we will define the problem. Finally, the proposed model will be elaborated in detail, including architecture design, feature extraction and node generation.

### Motivation

Currently, fraud detection still faces the problem of class imbalance and underrepresentation of features. The main reason is that fraudsters only account for a very small percentage of the overall fraudulent transactions, and Traditional GNN-based methods often fail to effectively capture fraud-related features, as these methods may obscure critical fraud-related features amidst a large volume of non-fraudulent data. In response, this paper proposes the use of subgraph-based techniques to focus on analyzing interactions typical of fraud rings or “small gangs”. This approach allows for the extraction of key features from subgraphs, which enables more precise detection of fraud details, thereby overcoming the limitations of broad feature aggregation typical in traditional GNNs and significantly improving the recognition of minority classes. Inspired by the SAT^37^ model, a structure-aware transformer model has been improved to enhance the feature extraction process. This model is specifically designed to preserve structural patterns within subgraphs, ensuring that key features are not lost during aggregation. Additionally, the scale and structural details of these subgraphs are dynamically adjusted to accurately reflect real-world transaction patterns, enhancing the authenticity and applicability of the features.

Further, to optimize our method, a targeted feature selection strategy has been adopted for generating new nodes within subgraphs, focusing on tightly linked features to create meaningful and representative new nodes that reflect potential fraudulent activities. Moreover, it has been specified how new edges should be linked, ensuring all newly created elements maintain logical and practical connections with existing data points, thereby maintaining the integrity of the graph structure and enhancing the reliability and relevance of the data.

### Problem definition

#### Definition 1

(Node embedding of graph data). The following defines the graph as $G=\left\{ V,E,A,X,C\right\}$. Where $V=\left\{ V_1,\ldots , V_N\right\}$ denotes a set of nodes. $E=\left\{ E_1, \ldots , E_H\right\}$ denotes a set of edge relations of H.

In graph *G*, $A\in H^{ n \times n}$ represents the adjacency matrix. $X\in H^{n\times d}$ denotes the node feature matrix, with $X[l,:]\in H^{1\times d}$ in *H* representing the features of node *l* and *d* being the feature dimension. $Z\in H^n$ contains the class information for the nodes in *G*. Throughout training, solely a subset $Z_J$ is accessible, containing labels for a subset $V_J$ of nodes.

There are a total of *m* classes, $\left\{ C_1,\ldots ,C_m\right\}$. $|C_i|$ is the size of class *i* and refers to the number of samples belonging to that class. The imbalance ratio, $\frac{mini(|Ci|)}{max_{i}(|Ci|)}$, is defined to measure the degree of class imbalance.

Given graph *G* with an imbalanced node class set, where labels are available only for a subset of nodes $V_J$, our objective is to develop a node classifier *f* capable of effective classification for both majority and minority classes, i.e.,

1 $$\begin{aligned} f(V,A,X)\rightarrow Z \end{aligned}$$

#### Definition 2

(GNN-based fraud detection). The GNN-based fraud detection task is defined on a multirelational unbalanced graph *G*. Node $v_i$ in *V* represents a network entity such as a device or a message comment. *C* is the label set of node *V*, with $c_i = 0$ for benign nodes and $c_i = 1$ for fraudulent nodes. The task of GNN-based fraud detection is to differentiate fraud nodes from benign nodes with in a graph *G*. Given the distinct characteristics of fraud nodes, this problem can be formulated as an imbalanced node classification problem on graph *G*.

### The proposed GTS

In this subsection, a detailed description of the GTS is given. The main idea of GTS is to use subgraph gnn extractor to acquire subgraph structural information of a node so that the feature representation carries both structural and feature information. The feature information is used as input to the transformer so that the obtained node features carry both structural and positional information. Synthesize the obtained node features into few oversampled nodes. The extracted node features are leveraged to generate synthetic minority nodes. Additionally, an edge generator is utilized to predict the connections among these synthesized nodes within the specified subgraph range. This process constructs an augmented balanced graph, which facilitates more efficient node classification.

**Figure 2:**
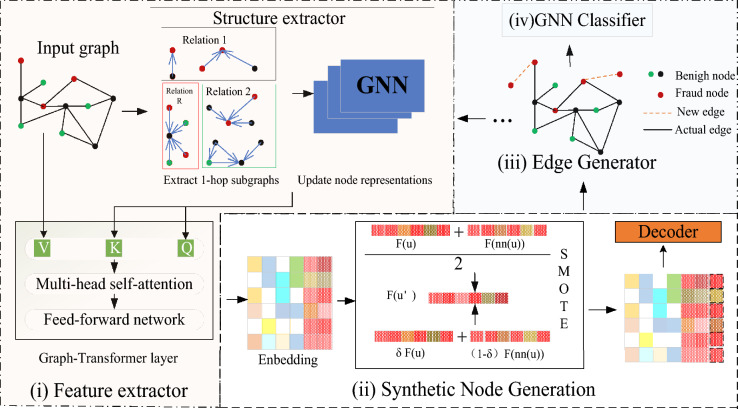
shows an overview of the proposed framework, the GTS consists of four main components. In the model framework, the structure extractor performs structural searches and feature aggregation on the input heterogeneous graph data according to different relationships, preparing feature enhancement for downstream tasks. In the model framework, each different type of relationship generates corresponding node representations and edge links.

Figure 2 shows an overview of GTS, and the GTS consists of four main components: (1) a feature extractor, which designed to learn node representations that capture both node attributes and graph structure information. By doing so, it augments the node feature representations, enriching them with additional context and relationships, which in turn enhances the performance of downstream tasks such as node classification; (2) a synthetic node generator, which synthesizes nodes with few classes by embedding node features obtained in (1) into feature space; (3)synthetic nodes in the subgraph range for synthetic nodes with a balanced class of augmented graph generating links; (4) The GNN-based classifier utilizes the augmented graph to classify nodes. It leverages the enriched node representations learned by the feature extractor to make accurate predictions. In the following sections, we will delve into the details of each component.

#### Feature extractor

In order to make the obtained node features more primitively expressive, a feature extractor that obtains both node features and graph structural information is introduced. In the search for similar node representations, not only the node features and labels but also the structural information are considered. In order to extract node features that are also structurally similar, a set of subgraphs centered on each node is introduced. The structure-aware extractor is defined as:

2 $$\begin{aligned} attn(u)=\sum _{v\in V}\frac{k_{g}\left( S_{G}(u),S_{G}(v)\right) }{\sum _{m\in V}k_{g}\left( S_{G}(u),S_{G}(m)\right) }f\left( x_{v}\right) \end{aligned}$$

where $S_{G}(u)$ denotes the subgraph centered on the node *v* associated with the node feature *X* in *G*, and $k_{g}$ can be the kernel of any comparative pair of subgraphs. This new self-attention mechanism does not only take into account the similarity of attributes, but also synthesizes the structural similarity between subgraphs, and thus it produces a more expressive representation of nodes. Moreover, it is isomorphic only for nodes with similar features and subgraph structures.

Next, the model Kg, which is fully expressive and computationally tractable, is presented:

3 $$\begin{aligned} k_{g}\left( S_{G}(v),S_{G}(u)\right) =k_{exp}\left( \varphi (v,G),\varphi (u,G)\right) \end{aligned}$$

where $\varphi (u,G)$ denotes the structure extractor which extracts the vector representation of the subgraph centered at *u* with node feature *X*. We use *h* subtrees as GNN extractors then:

4 $$\begin{aligned} \varphi (u,G))=GNN_{G}^{h}(u) \end{aligned}$$

$GNN_{G}^{h}(u)$ denotes an arbitrary GNN model, where *h* denotes the number of extracted layers. In practice, *a* small value of *h* has led to good performance without suffering from over-smoothing or over-squeezing. After getting the representation of the node features, the message passing and fusion process is set up:

5 $$\begin{aligned} h_{v}^{1}=\delta \left( W^{1}\cdot CONCAT\big (P[v,:],P\cdot A[:,v]\big )\right) \end{aligned}$$

Let *P* represent the input node attribute matrix, where *P*[*v*,  : ] denotes the attributes of node *V*. *A*[ : , *v*] denotes the *v*-th column in the adjacency matrix, capturing the connections of node *V* with other nodes. The embedding of node *V* is denoted as $h_{v}^{1}$, and $W^{1}$ is a weight parameter. The activation function, such as ReLU, is represented by $\delta$.

#### Synthetic node generation

After obtaining the features of the nodes through the feature extractor, we try to synthesize a new minority class node representation by synthesizing the minority oversampling. The previous intention is mainly achieved by the SMOTE algorithm, which can preserve the features of the original data and comprehend the distribution of real data to generate new minority class samples. This is achieved through interpolation between existing minority class samples, effectively expanding the minority class representation while maintaining data integrity. In addition by increasing the number of samples of minority classes, the distribution of classes is balanced and the model’s ability to recognize minority classes is improved. Specifically, the Smote algorithm consists of the following steps:

1. Selecting a sample of the minority class: Select a sample $h_{u}^{1}$ from the minority class as a starting point, labeled $Z_{u}$.
2. Selecting Neighboring Samples: Use the Euclidean distance in the embedding space to select a nearest-neighbor sample for that minority class sample:
  6 $$\begin{aligned} nn(u)=\underset{v}{argmin}\left\| h_{v}^{1}-h_{u}^{1}\right\| ,\quad s.t.\quad Z_{v}=Z_{u} \end{aligned}$$
  *nn*(*u*) is the nearest neighbor of *v* in the same class.
3. Generating synthetic samples: In the selected few class samples, when the node features are very similar, the average of two node features is taken:
  7 $$\begin{aligned} h_{v'}^{1}=\frac{h_{v}^{1}+h_{nn(v)}^{1}}{2} \end{aligned}$$
  Otherwise, a random variable is used to select the proportion of nodes:
  8 $$\begin{aligned} h_{v'}^{1}=(1-\delta )\cdot h_{v}^{1}+\delta \cdot h_{nn(v)}^{1} \end{aligned}$$
  where a is $\delta$ random variable, uniformly distributed in the range [0,1].

Given that $h_{v}^{1}$ and $h_{nn(v)}^{1}$ share the same class label and exhibit close proximity in the feature space, it is reasonable to assume that the synthetic node $h_{v'}^{1}$ generated through interpolation should also belong to the same class. This process enables the generation of labeled synthetic nodes. A hyperparameter controls the number of samples generated for each class, allowing the number of synthetic samples for minority classes to be increased, thereby balancing the class distribution.

#### Edge generator

The above work accomplishes the generation of nodes for a small number of classes. However, the newly generated nodes are independent and no edges are connected to the original graph *G*. To ensure the authenticity of the generated nodes, an edge generator is introduced in the selected features to model the newly generated edges. This edge generator learns the representation of the edges and predicts the neighborhood information of the new nodes within the subgraph of the selected features. The feature representation of the edge is generated by splicing the connected node features. The edge features are mapped to between 0 and 1 by a Sigmoid function that predicts the relationship of edges between two nodes:

9 $$\begin{aligned} Q\left( e_{ij}=1|h_{i},h_{j}\right) =\delta \left( W\left[ h_{i},h_{j}\right] +b\right) \end{aligned}$$

where $e_{ij}$ represents the edge connecting node *i* and node *j*. The feature representations of node *i* and node *j* are denoted by $h_{i}$ and $h_{j}$, respectively. The Sigmoid activation function is represented by $\delta (x)$, *W* is the weight matrix, and $[h_{i},h_{j}]$ denotes the concatenation of node features.

The loss function employed to train the edge generator is given by:

10 $$\begin{aligned} L_{edge}=\Vert Q-A\Vert _{P}^{2} \end{aligned}$$

where *A* is the initial adjacency matrix. By setting the threshold $\eta$, the predicted edges of the new node are added to the augmented adjacency matrix:

11 $$\begin{aligned} \widetilde{A}[{v}',u]={\left\{ \begin{array}{ll} 1,&{}\quad if \; E_{{v}',u}> \eta \\ 0,&{}\quad otherwise \end{array}\right. } \end{aligned}$$

where $\widetilde{A}$ is the oversampled adjacency matrix and it is sent to the classifier.

#### GNN classifier

After the above work an augmented balanced graph is obtained $$\widetilde{G}=\begin{Bmatrix} \widetilde{V},&\widetilde{A},&\widetilde{X} ,&\widetilde{T} \end{Bmatrix}$$. $\widetilde{A}$ denotes the augmented matrix, $\widetilde{X}$ denotes the augmented node features, $\widetilde{V}$ denotes the augmented label set and $\widetilde{T}$ denotes as the final augmented node set by concatenating ${T}^{1}$ (embedding of real nodes) with the embedding of the synthetics nodes. We train the GNN classifier on graph $\widetilde{G}$. An additional graph block is used with an additional linear layer for node classification:

12 $$\begin{aligned} h_{v}^{2}= & {} \delta \left( W^{2}\cdot CONCAT\left( h_{v}^{1},\widetilde{T}\cdot \widetilde{A}[:,V]\right) \right) \end{aligned}$$

13 $$\begin{aligned} Q_{v}= & {} softmax\left( \delta \left( W^{c}\cdot CONCAT\left( h_{v}^{2},T^{2}\cdot \widetilde{A}[:,V]\right) \right) \right) \end{aligned}$$

where $T^{2}$ represents the node representation matrix of the second illustrated block and *W* as the weight parameter, the probability distribution of node *v* over the class labels is denoted by $Q_{v}$. Optimization is achieved through cross-entropy loss, which is calculated as follows:

14 $$\begin{aligned} L_{node}=\sum _{u\in \widetilde{v}_{L}}\sum _{c}\left( 1\left( Z_{u}==c\right) \right) \cdot log\left( Q_{v}[c]\right) \end{aligned}$$

And during the testing phase, the predicted class label for node *v*, denoted as ${Z}'_{v}$, is assigned to the class with the highest probability,

15 $$\begin{aligned} {Z}'_{v}= \underset{c}{argmax}Q_{v,c} \end{aligned}$$

The model’s performance is contingent upon the quality of feature extraction and generated edges. The overarching objective function of GTS can be expressed as:

16 $$\begin{aligned} \underset{\theta ,\phi ,\varphi }{min}L_{node}+L_{edge} \end{aligned}$$

where $\theta$, $\phi$, $\varphi$ are the parameters of feature extractor, edge generator and node classifier respectively.

#### Training algorithm

**Algorithm 1:**
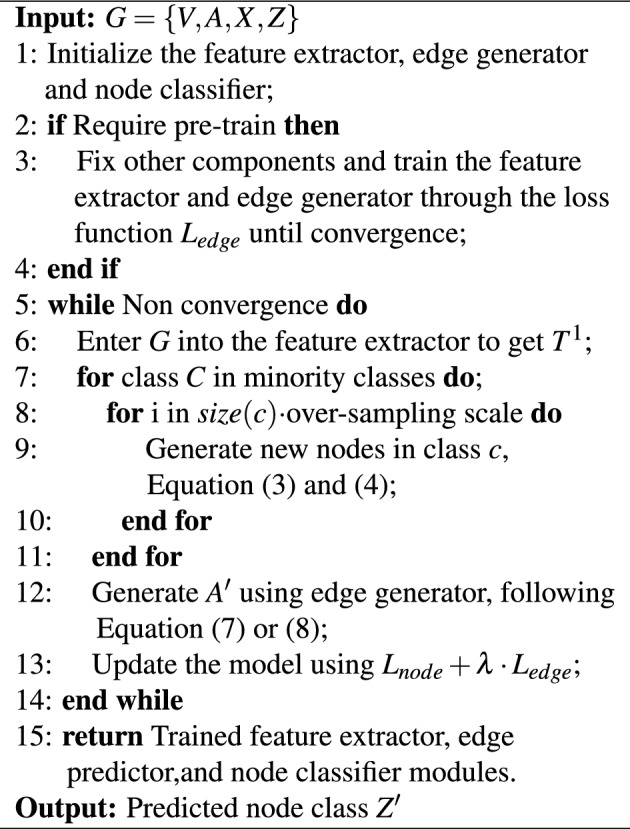
Full training algorithm

## Experiments

In this section, experiments on the proposed GTS model are conducted on a GNN-based fraud detection dataset, with the aim of addressing the following research questions:

- Is GTS a state-of-the-art method in graph-based fraud detection tasks?
- How do the GTS key modules benefit the end result?
- How does the GTS perform relative to different training parameters?

### Experimental setup

#### Datasets

We conducted experiments on two popular real-world datasets, YelpChi^9^ and Amazon^38^, to evaluate the performance of GTS. Table 1 lists the statistics of the two datasets.

**Table 1:** Statistics of the two fraud detection datasets.

Dataset	#Node (Fraud%)	#Edge	Relations	#Relations
YelpChi	45,954 (14.5%)	3,846,979	R–U–R	49,315
			R–S–R	3,402,743
			R–T–R	573,616
Amazon	11,944 (9.5%)	4,398,392	U–P–U	175,608
			U–S–U	3,566,479
			U–V–U	1,036,737

The YelpChi dataset consists of hotel and restaurant reviews filtered (spam) or recommended (legit) by Yelp. We obtained 32 manual features as raw features for reviews in the YelpChi dataset. After preprocessing, the final dataset contains 45,954 reviews (14.5% spam).

The Amazon dataset consists of reviews of products under the musical instruments category. Users in this dataset are labeled as legitimate (using more than 80% of the votes) and fraudulent (less than 20% of the votes), and the nodes in the graph are users with 100-dimensional features, with 25 manual features from^39^ as the original user features.

As in earlier work^25^, a classification task is performed to identify whether a user or a review is fraud or not.

#### Baselines

To ascertain the efficacy of the GTS method in fraud detection tasks, we compared it with both traditional GNN models and several state-of-the-art GNN-based fraud detection models. The main ideas of each baseline method are described as follows:

GCN^40^: The model achieves semi-supervised learning through a first-order approximation of spectral graph convolutions.

GAT^20^: This model leverages masked self-attention layers to address the limitations of graph convolution methods.

GraphSAGE^19^: This model achieves node embeddings by sampling and aggregating node feature information.

GraphConsis^41^: This model is a graph neural network framework that addresses context inconsistency, feature in inconsistency, and relation inconsistency.

CARE-GNN^25^: This model enhances the GNN aggregation process to resist feature and relation camouflages by using a label-aware similarity measure, reinforcement learning to select optimal neighbors, and aggregation across different relations.

PC-GNN^15^: This model addresses class imbalance in graph-based fraud detection by using label-balanced sampling and neighborhood sampling to aggregate information.

AO-GNN^16^: This model addresses the issue of label imbalance and noisy edges in GNN-based fraud detection by maximizing the AUC metric and implementing an edge pruning strategy.

LGM-GNN^28^: This model addresses the problem of neglecting global information in fraud detection through a local and global aware memory-based graph neural network framework.

$\mathrm{H}^2$-FDetector^23^: This model addresses the issue of existing GNN-based fraud detection methods ignoring heterphilic connections by using a new aggregation srategy to handle both homophilic and heterophilic interactions.

HHLN-GNN^35^: This model addresses the imbalance and complexity of financial transacion data by using subgraph generators and neighborhood samplers for category imbalance, and a self-attentive module to differentiate between homogeneous and heterogeneous connections.

#### Experimental settings

For all baseline and GTS models, we performed a 10-run experiment to evaluate them using the final mean and standard deviation. The number of attentional heads was set to 8 and the number of layers was set to 6. We divided the dataset into training, validation, and testing sets, which accounted for 40%, 20%, and 40%, respectively. Our experiments use Adam as an optimizer to optimize the parameters of GTS. For the training process, we initialize the learning rate to 0.01, set the weight decay to 0.001, and select a hidden layer dimension of 64 for the node features. Additionally, we employ a dropout rate and a positive sample oversampling rate, both set to 0.5. The number of training ephemerides for both datasets is 200 to ensure that the model learns the data features adequately. In the YelpChi and Amazon datasets, batch sizes of 256 and 1024 are used to perform gradient descent updates, respectively. When performing subgraph extraction, we set the hop count to 2. We choose cross-entropy loss as the loss function to measure the gap between the model predictions and the true labels.

In the comparative experiments, the GCN, GraphSAGE, and GAT models were implemented using DGL^42^, while methods like GraphConsis, CARE-GNN, and PC-GNN were implemented using the public source code provided by the authors with parameters recommended in their respective papers. The entire code for GTS was developed using Pytorch 1.8.0 and Python 3.9, and all experiments were conducted on two NVIDIA A100 GPUs.

#### Evaluation metrics

In order to evaluate the unbalanced dataset without bias, we use three metrics, F1-macro, AUC and GMean, to measure the performance of all methods.

F1-macro combines two metrics, Precision and Recall, which has the advantage of treating each category equally and is not affected by uneven distribution of categories.The formula for F1-macro is as follows:

17 $$\begin{aligned} F1{\text{-}}macro=\frac{1}{N} \sum _{i=1}^{N}F1_{i} \end{aligned}$$

where N denotes the total number of categories.

AUC is the area under the ROC curve, and its value ranges from 0 to 1. The larger the AUC value, the better the model performance. Its advantage is that it is not sensitive to the problem of category imbalance, even in the case of the proportion of positive and negative categories in the sample, AUC can also objectively reflect the performance of the model. the formula for calculating AUC is as follows:

18 $$\begin{aligned} AUC=\frac{\sum _{u\in U^{+}}rank_{u}-\frac{|u^{+}|\times (|u^{+}|)+1}{2}}{|u^{+}|\times |u^{-}|} \end{aligned}$$

Here, $u^{+}$ and $u^{-}$ denote the minority and majority class sets in the test set, respectively. $rank_{u}$ denotes the ranking of node u by its prediction score.

GMean, which is the geometric mean of model precision and recall. In unbalanced datasets, G-Mean is usually more reliable than Accuracy because it takes into account the balance between positive and negative categories.The formula for GMean is as follows:

19 $$\begin{aligned} GMean=\sqrt{TPR\cdot TNR} \end{aligned}$$

### Performance comparison

Fraud detection performance on two datasets, compared with popular benchmark methods. The evaluation metrics include the area under the roc curve (AUC), macro-averaged F1 score (F1-macro), and geometric mean (GMean).

The performance of GTS is compared below with all the previously mentioned baselines. The results are shown in Table 2 and the following conclusions are observed.

**Table 2:** Fraud detection performance on two datasets, compared with popular benchmark methods.

Method	Datasets	YelpChi			Amazon		
	Metric	AUC	F1-macro	GMean	AUC	F1-macro	GMean
Baselines	GCN	0.5983 ± 0.0049	0.5620 ± 0.0067	0.4365 ± 0.0262	0.8369 ± 0.0125	0.6408 ± 0.0694	0.5718 ± 0.1951
	GAT	0.5715 ± 0.0029	0.4879 ± 0.0230	0.1659 ± 0.0789	0.8102 ± 0.0179	0.6464 ± 0.0387	0.6675 ± 0.1345
	GraphSage	0.5439 ± 0.0025	0.4405 ± 0.1066	0.2589 ± 0.1864	0.7589 ± 0.0046	0.6416 ± 0.0079	0.5949 ± 0.0349
	GraphConsis	0.6983 ± 0.0302	0.5870 ± 0.0200	0.5857 ± 0.0385	0.8741 ± 0.0334	0.7512 ± 0.0325	0.7677 ± 0.0486
	CARE-GNN	0.8178 ± 0.0014	0.6400 ± 0.0230	0.7395 ± 0.0130	0.9586 ± 0.0014	0.8956 ± 0.0077	0.9030 ± 0.0044
	PC-GNN	0.7619 ± 0.0292	0.6332 ± 0.0094	0.6791 ± 0.0359	0.9067 ± 0.0112	**0.8990** ± 0.0073	0.8962 ± 0.0018
	AO-GNN	0.8805 ± 0.0008	0.7042 ± 0.0051	0.8134 ± 0.0232	0.9640 ± 0.0020	0.8921 ± 0.0045	0.9096 ± 0.0105
	$\mathrm{H}^2$-FDetector	0.8265 ± 0.0058	0.6489 ± 0.0456	0.7515 ± 0.0096	0.9191 ± 0.0100	0.7927 ± 0.0206	0.8394 ± 0.0577
	LGM-GNN	0.8241 ± 0.0009	0.6717 ± 0.0118	0.7604 ± 0.0016	0.9609 ± 0.0000	0.8625 ± 0.0002	0.9127 ± 0.0001
	HHLN-GNN	0.8425 ± 0.0314	0.6959 ± 0.1230	0.7454 ± 0.3000	0.9171 ± 0.0014	0.8804 ± 0.0070	0.9083 ± 0.0032
Ours	GTS	**0.8915** ± 0.0102	**0.7228** ± 0.0007	**0.8159** ± 0.0319	**0.9685** ± 0.0015	0.8671 ± 0.0284	**0.9164** ± 0.0062

We report the results of the area under the ROC curve (AUC), macro average of F1 score (F1-macro), GMean.

Significant values are in [bold].

DR-GCN adds conditional adversarial regularization layer and latent distribution alignment regularization layer to gnn to address the graph data imbalance problem. PC-GCN and AO-GCN are two state-of-the-art graph-based fraud detection methods, which also focus on the label imbalance problem. In contrast, GTS uses subgraph extractor to aggregate neighborhood information, uses feature extraction with SAT, and finally uses synthetic few oversampling to balance the nodes. The experimental results show that GTS achieves better results except for the F1-macro for the Amazon dataset. Besides, GraphConsis, CARE-GNN are excellent models for GNN-based fraud detection. They attempted to leverage a node distance metric for neighborhood selection in addressing the forgery problem. However, due to insufficient optimization of the node distance metric function, they were unable to achieve significant performance improvements.

GCN, GAT and GraphSAGE are the classical GNN models. GCN mainly focuses on the first-order neighbor information of nodes, which limits the ability to capture the global features of nodes. Meanwhile, for graph data with unbalanced labels, GCN may suffer from the underfitting problem to a few categories, which affects the classification performance. Since GAT introduces an attention mechanism that requires a large number of parameters, but the minority class lacks sufficient data to participate in the training of the model, GAT has the lowest score. GraphSAGE operates with a fixed-size neighborhood. For nodes with larger neighborhoods, this approach leads to a loss of information, hindering its performance. Consequently, GraphSAGE demonstrates inferior performance compared to PC-GNN.

### Ablation study

The model involves several key components, and in order to assess the impact of each component on the overall performance, we performed ablation experiments. In particular, the experiment was constructed by eliminating the tranformer (GTS$\backslash$CT) component and eliminating the SMOTE (GTS$\backslash$CS) component, and the results are shown in Table 3.

**Table 3:** The testing performance for different variants of GTS.

Method	Datasets	YelpChi			Amazon		
	Metrics	AUC	F1-macro	Gmean	AUC	F1-macro	Gmean
GTS\CT		0.7125 ± 0.0020	0.5153 ± 0.0107	0.6342 ± 0.0075	0.8971 ± 0.0408	0.7646 ± 0.0027	0.8392 ± 0.0137
GTS\CS		0.7518 ± 0.0216	0.6340 ± 0.0151	0.7354 ± 0.0506	0.9106 ± 0.0339	0.7913 ± 0.0135	0.8637 ± 0.0033
GTS		**0.8915** ± 0.0102	**0.7228** ± 0.0007	**0.8059** ± 0.0309	**0.9685** ± 0.0015	**0.8671** ± 0.0284	**0.9164** ± 0.0062

Significant values are in [bold].

Clearly, the performance of both variants is significantly degraded compared to the full GTS model, proving their effectiveness for GTS. Also the performance of GTS$\backslash$CT is better than that of GTS$\backslash$CS, which proves the effectiveness of the structure-aware graph-based transformers for feature extraction.

Moreover, comparing GTS with GTS$\backslash$CS , they still have a significant decrease in performance. This demonstrates that achieving balance by synthesizing few classes of nodes helps in fraud detection node classification. In summary, removing any of the components degrades the performance of GTS, which proves that both transformer and SMOTE are effective in graph-based fraud detection methods.

### Sensitive analysis

**Figure 3:**
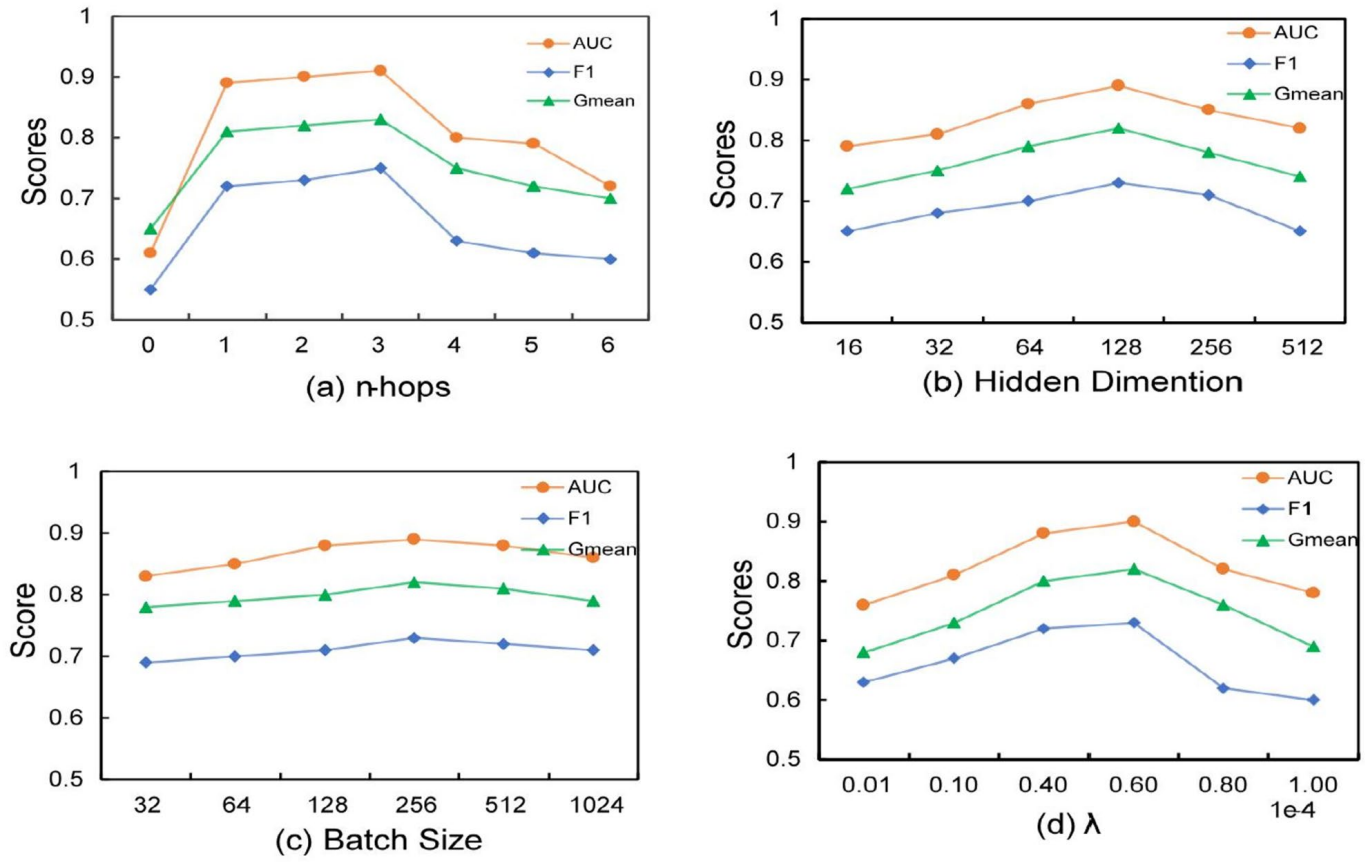
Analysis of model sensitivity by subgraph extraction hops count, hidden dimension, batch size and hyperparameter $\lambda$, respectively.

In this section, we investigate the sensitivity of the model parameters by varying the hidden dimension, batch size and hyperparameter $\lambda$. The experimental results for the fraud detection dataset are shown in Fig. 3.

The key contribution of a structure-aware graph transformer is the ability to explicitly incorporate structural information into the self-attention mechanism. The following changes the hop count *n* of the graph transformer from 1 to 6, showing how the size of *n* affects the performance ($n=0$ corresponds to a normal transformer without structure-awareness).

As can be seen from Fig. 3a, the optimal performance the optimal performance of the *n*-subgraph extractor is around $n=3$. When *n* is increased beyond $n=4$, the performance of the *n*-subgraph decreases. The results show that when the graph is too large, it is prone to over-smoothing; while when the graph is too small, there is not enough information in the aggregated domain and over-squeezing occurs.

Figure 3b shows that gradually increasing hidden dimension from 16 to 128 the model shows a continuous increase in performance. The peak of relative performance is reached at a dimension of 128. This suggests that the choice of embedding dimension is critical to model performance. With too small a dimension, the model may not be able to learn the features adequately, leading to underfitting, while too large a dimension may increase the model complexity, leading to overfitting.

Figure 3c shows that our model performs best when the batch size is set to 64 or 256. Considering the training efficiency of the model, we set the batch size to 256.

The following tests the sensitivity of the GTS to it via the hyperparameter $\lambda$. The hyperparameter $\lambda$ is a trade-off between model complexity and loss. We set $\lambda$ at $$\begin{Bmatrix}1e{-}6,&1e{-}5,&4e{-}5,&6e{-}5,&8e{-}5,\\ {}&1e{-}4\end{Bmatrix}$$. Each experiment is performed on the Yelp dataset. The results are shown in Fig. 3d, where it can be seen that as $\lambda$ increases, the performance first improves and then decreases. If the $\lambda$ value is too large, the performance will drop significantly. This is because too large a $\lambda$ may cause the model to under-fit on the training data, reducing the model’s fitting ability. Generally, it works better between $4e{-}6$ and $8e{-}5$.

**Figure 4:**
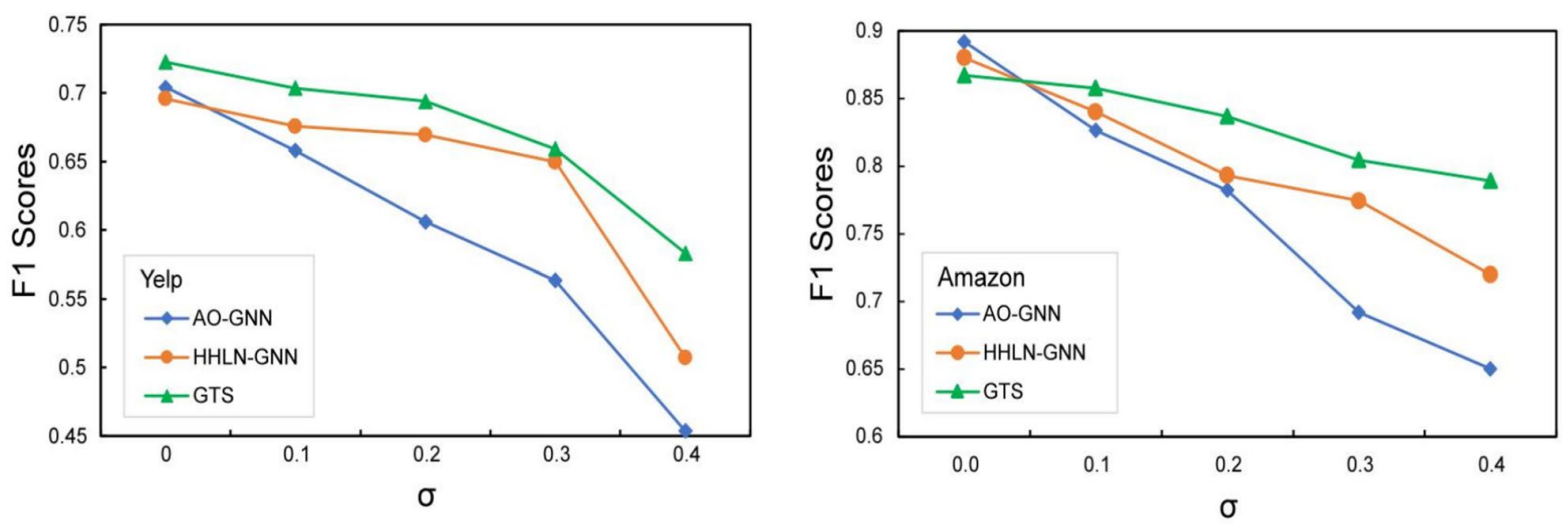
Analysis of model robustness to noise, represented by $\sigma$, on Yelp and Amazon datasets.

The noise comparison experiments presented illustrate the performance differences among various GNN methods (AO-GNN, HHLN-GNN, GTS) as noise levels increase in Fig. 4, where noise intensity is represented by the horizontal axis, $\sigma$. On the Yelp dataset, AO-GNN’s performance rapidly deteriorates with increasing noise levels, while HHLN-GNN experiences a slower decline but also shows a significant decrease in performance when noise reaches 0.4. In contrast, GTS demonstrates greater robustness, with only minor reductions in F1 scores even as noise increases. On the Amazon dataset, AO-GNN exhibits the quickest decline in performance, particularly when noise increases from 0.2 to 0.3. Conversely, HHLN-GNN, although also showing a performance decline, does so at a more gradual pace. GTS continues to maintain better performance, proving its stability across different noise levels. These results highlight the advantage of GTS in high-noise environments.

**Figure 5:**
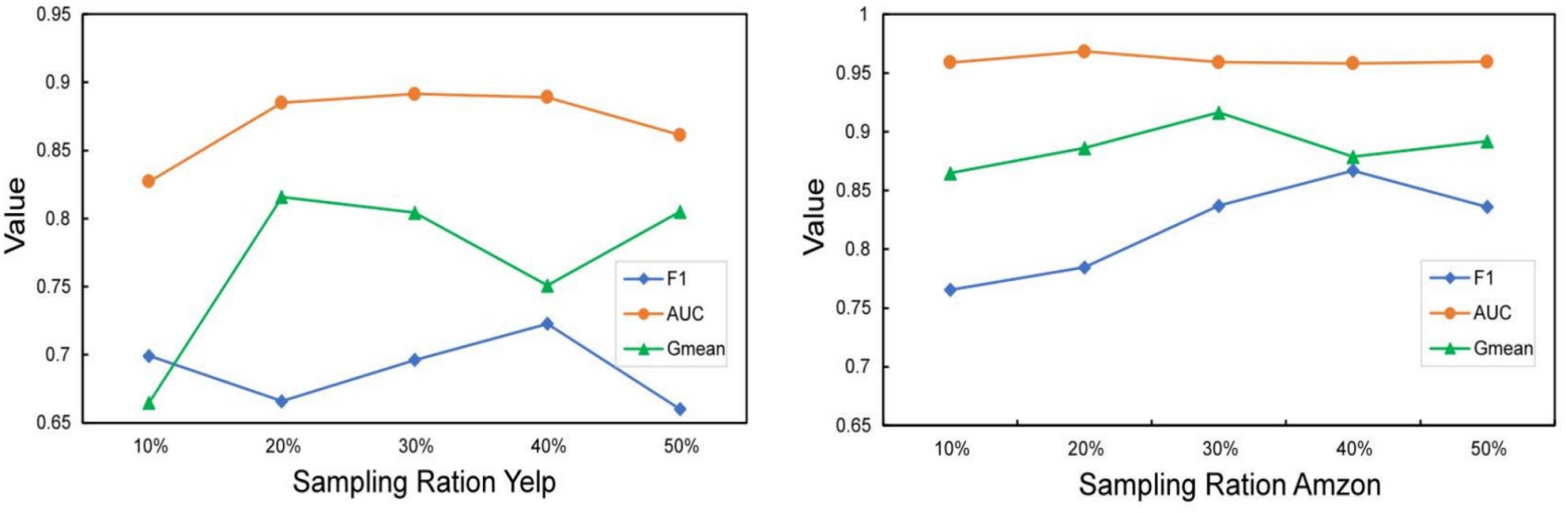
Analysis of model robustness to sampling ration on Yelp and Amazon datasets.

The Fig. 5 demonstrates the impact of varying oversampling ratios on the Yelp and Amazon datasets using the GTS method. For the Yelp dataset, AUC values steadily rise from 10 to 30%, then slightly decline at 40%, indicating that oversampling benefits model stability. The F1 score peaks at 30% and 40% before declining, suggesting that excessive oversampling could lead to overfitting. GMean peaks at 20% and then diminishes, indicating that moderate oversampling enhances minority class recognition, but excessive levels reverse this effect. On the Amazon dataset, AUC values remain stable from 10 to 50%, with the F1 score slightly decreasing after 40%, implying that appropriate oversampling improves overall performance, whereas excessive oversampling is detrimental. Fluctuations in GMean after 30% highlight the model’s sensitivity to oversampling levels.

## Conclusion

In this paper, we propose a GNN-based method called GTS to deal with the class imbalance problem in fraud detection using a combination of node-centered structure-aware Transformer and SMOTE in order to obtain enriched node features and generate minority class nodes. Specifically, we adopt a node-centered subgraph structure extraction to ensure the authenticity of the node synthesis for the few classes that follow as well as the edge generation. In the SMOTE module, two strategies are used to handle the similarity between the extracted features, which significantly improves the reliability of the synthesized nodes. At the same time, edges are provided for the newly generated nodes within the subgraph of the feature extraction to ensure the similarity of the nodes. Finally, the number of samples generated for each class is controlled using hyperparameters to reach the final equilibrium.Extensive experiments were conducted on two real datasets and the results proved the effectiveness of GTS. For future work, in addition to structure-aware node extraction, learning new graph structures for unbalanced graphs based on dynamic graph length and short information dependencies may be a promising direction for GNN-based fraud detection.

## Data Availability

All data generated or analyzed during this study are included in this published article. In the proposed work, two datasets have been used, namely YelpChi and Amazon. For your ready reference, the link for two datasets are provided below: 1.YelpChi: https://paperswithcode.com/dataset/yelpchi 2.Amazon: https://paperswithcode.com/dataset/amazon-fraud
